# TAN-FGBMLE: Tree-Augmented Naive Bayes Structure Learning Based on Fast Generative Bootstrap Maximum Likelihood Estimation for Continuous-Variable Classification

**DOI:** 10.3390/e27121216

**Published:** 2025-11-28

**Authors:** Chenghao Wei, Tianyu Zhang, Chen Li, Pukai Wang, Zhiwei Ye

**Affiliations:** 1 School of Computer Science, Hubei University of Technology, Wuhan 430068, China; chenghao.wei@hbut.edu.cn (C.W.);; 2 Hubei Provincial Key Laboratory of Green Intelligent Computing Power Network, Hubei University of Technology, Wuhan 430068, China

**Keywords:** Tree-Augmented Naive Bayes, class-conditional mutual information, complex density estimation, generative model, bootstrap, maximum likelihood estimation

## Abstract

Tree-Augmented Naive Bayes (TAN) is an interpretable graphical structure model. However, its structure learning for continuous attributes depends on the class-conditional mutual information, which is sensitive to one-dimensional or two-dimensional density estimation. Accurate estimation is challenging under complex distributions such as multi-peak, long-tailed and heteroscedastic cases. To address this issue, we propose a structure learning method for TAN based on Fast Generative Bootstrap Maximum Likelihood Estimation (TAN-FGBMLE). FGBMLE consists of two stages of work. In the first stage, resampling weights and random noise are input into a network generator to rapidly produce candidate parameters, efficiently covering the latent density space without repeated independent optimization. In the second stage, optimal mixture weights are estimated by maximum likelihood estimation, assigning appropriate contributions to each candidate component. This design enables fast and accurate complex density estimation for both single and joint attributes, providing reliable computation of class-conditional mutual information. The TAN structure is then constructed using Prim’s maximum spanning tree algorithm. Experiments show that our estimation method attains higher fitting accuracy and lower runtime compared with traditional nonparametric estimators. By using open-source datasets, the TAN-FGBMLE achieves superior accuracy and recall compared to classic methods, demonstrating good robustness and interpretability. On publicly available real air quality data, it has a high classification result and produces graph structures that more accurately capture dependencies among continuous attributes.

## 1. Introduction

Bayesian approaches are crucial in statistics and machine learning, offering a cohesive theoretical structure for modeling and inferring uncertainty [1]. They have been widely applied in fields such as engineering monitoring [2], control theory [3], and medical diagnosis [4]. Their core concept is to update beliefs about underlying parameters or structures by combining prior knowledge with data evidence, thereby enabling systematic reasoning from data. The emergence of probabilistic graphical models has provided a powerful tool for discovering causal or dependent relationships in complex systems. Probabilistic graphical models, by representing conditional independence among variables with graph structures, allow for the modeling and inferring complex high-dimensional distributions.

The Naive Bayes (NB) model has achieved great success in practice due to its simplicity and efficiency [5]. However, NB relies on a strict conditional independence assumption, namely that features are independent of each other given a class label. While the assumption greatly simplifies computation, it often does not hold true across real-world datasets. Dependencies between features often exist. Ignoring these dependencies can lead to classifier bias, insufficient expressiveness, and poor predictive performance [6]. To address this limitation, researchers have proposed the TAN model [7]. By upholding NB’s simplicity, the TAN model allows attribute interdependencies to be encoded in a tree structure, thereby balancing computational feasibility with model accuracy. The TAN model is a theoretical extension of NB, exhibiting notable benefits in areas like text classification, genomics, and medical diagnosis.

The core of TAN lies in learning dependency structures. The key step in learning is estimating class-conditional mutual information (CMI) [8]. Mutual information quantifies the strength of the statistical dependencies between feature pairs and directly determines the selection of edges in the TAN structure. Large biases in the CMI estimation can lead to incorrect representation of dependencies, compromising classification accuracy and interpretability. Therefore, achieving stable and accurate CMI estimation is a primary challenge, especially in continuous attribute conditions [9].

The calculation of mutual information for continuous variable relies on one-dimensional and two-dimensional class-conditional density estimation. However, real-world data distributions are often far from simple unimodal and symmetric cases. They may exhibit multimodal structures, long-tailed distributions, heteroskedasticity, and severe skewness. These complexities present substantial obstacles to the effectiveness of traditional density estimation methods [10]. Kernel density estimation (KDE) has the advantages of being nonparametric and consistent in low dimensions [11], but suffers from the curse of dimensionality and local overfitting in high dimensions when the data distribution is complex. Finite Gaussian Mixture Models (GMMs) can approximate complex distributions to a certain extent, but their sensitivity to the number of mixture components and their lack of adaptability to non-Gaussian distributions [12].

To overcome these shortcomings, hybrid approaches combining bootstrapping with generative modeling have gained increasing attention. Bootstrapping, a classic resampling technique, effectively captures sample uncertainty and improves estimation robustness [13]. Generative models excel at capturing the complex structure of the underlying distribution. Combining these two approaches could potentially achieve a balance between computational efficiency and estimation accuracy, overcoming the limitations of traditional methods. However, such approaches come with very high computational cost. Every resampling step requires a complete optimization of the likelihood function. In high-dimensional or large-sample problems, this repeated optimization becomes prohibitively expensive, making traditional bootstrap methods inefficient for practical applications.

To alleviate this computational burden, we propose a computational strategy based on the neural network generative process, called the Fast Generative Bootstrap Maximum Likelihood Estimator (FGBMLE). This method significantly reduces the computational cost and theoretically retains the expressive power and flexibility of bootstrap resampling. Our main contributions are as follows:

- This method constructs an optimization framework based on a neural network generator, thereby avoiding the high computational cost of repeatedly optimizing weight combinations in the traditional bootstrap. Instead of relying solely on resampling weights, the generator also incorporates an additional source of randomness. The combination of resampling information and stochastic perturbations allows the model to capture the essential representation of the optimization problem. By leveraging the expressive capacity of neural networks, this strategy enables the generator to flexibly adapt to complex distributional characteristics, substantially improving both the efficiency and accuracy of density estimation.
- Unlike traditional bootstrap methods, which require a complete optimization of the likelihood function at each resampling step, our approach condenses this repetitive and costly procedure into a single, efficient computation. This paper proposes a novel two-stage algorithm for the FGBMLE estimation process. In the first stage, the neural generator rapidly produces a set of candidate parameters that cover the potential distribution space by leveraging resampling information and additional randomness. In the second stage, maximum likelihood estimation is performed on this limited set to obtain the optimal configuration of mixture weights.
- The proposed FGBMLE is applied to TAN structure learning. Compared with traditional KDE and GMMs, FGBMLE has greater adaptability and stability in univariate and bivariate density estimation, enabling more reliable calculation of class-conditional mutual information, and thus the optimized TAN obtains more reasonable dependency structures. It significantly improves the stability of mutual information estimation and the reliability of structure learning.

In this work, the FGBMLE is proposed as a general two-stage framework for flexible density estimation and latent mixture modeling. The TAN structure learning is then chosen as a representative and practically important application of this framework, because it requires accurate class-conditional marginals and bivariate densities as core building blocks. Throughout the paper, we first develop and validate FGBMLE as a general estimator on synthetic density estimation tasks, and then instantiate it within TAN to obtain the proposed TAN-FGBMLE classifier. In this sense, TAN is not the sole goal of the method, but rather a concrete probabilistic graphical model that allows us to demonstrate how FGBMLE can be embedded into structure learning and classification.

The paper consists of the following parts. In Section 2, we provide a detailed discussion about the relevant research works. In Section 3, we describe in detail the complete structure learning process of the TAN-FGBMLE method. In Section 4, we carry out a range of experimental works for evaluating our method. Finally, in Section 5, we present the conclusion that summarizing the our work and outlining the potential future work.

## 2. Preliminaries

### 2.1. Latent Mixture Models and Semiparametric Estimation

Mixture models have long been a fundamental tool in statistics and machine learning for capturing heterogeneous or multimodal distributions. They provide a flexible framework for representing samples generated by latent variables. They have been extensively studied in both parametric and nonparametric settings.

To illustrate this framework, we use a mixed generation model to express the distribution characteristics of the observed data. Assuming that observation $Y=\left\{y_{1},y_{2},\ldots ,y_{n}\right\}\in R^{n}$ comes from the latent mixture model, we assume that the generation process of each $y_{i}$ is affected by an unobserved latent variable $\theta_{i}$, and these latent variables obey an unknown mixture density function π(θ).

The latent variable $\theta_{i}$ is independently sampled from the mixture density distribution π(θ). Then, given $\theta_{i}$, the observation data $y_{i}$ is generated according to the known observation model $f(y_{i}|\theta_{i})$. The model process can be expressed as the following equation.

(1) $$\theta_{i}∼\pi \left(\theta \right),\;y_{i}|\theta_{i}∼f\left(y_{i}|\theta_{i}\right),\;\forall i\in \left\{1,2,\ldots ,n\right\}.$$

Here, the observation model $f(y_{i}|\theta_{i})$ is a known conditional probability distribution [14], such as the normal distribution, Poisson distribution, or Gamma distribution, and π(θ) is the target function we want to estimate under the parameter conditions. After integrating the latent variable $\theta_{i}$, we can obtain the marginal distribution of $y_{i}$, which is in the form of the following equation.

(2) $$y_{i}∼\int f\left(y_{i}|\theta_{i}\right)d\pi \left(\theta_{i}\right)\;\forall i\in \left\{1,2,\ldots ,n\right\}.$$

This formula describes a typical mixed model structure. For each observation $y_{i}$, it is not generated by fixed parameters, but by a set of potential models $f(y_{i}|\theta_{i})$ with π(θ) as the mixing weight. It can be seen that π(θ) describes the diversity of the potential structure. It is the core object for our subsequent statistical inference and modeling.

To estimate the mixture density π(θ), we use semiparametric maximum likelihood estimation. Unlike classical mixture models, which assume a parameterized form with a finite number of components, we do not make a priori assumptions about the specific structure of π. Instead, we directly search for the optimal solution among the set of all probability density functions. We estimate π by maximizing the log-marginal likelihood function of the observed samples.

(3) $$\hat{\pi }=\underset{\pi \in \Pi }{\arg\max}\log p\left(y_{1},y_{2},\ldots ,y_{n};\pi \right)=\underset{\pi \in \Pi }{\arg\max}\sum_{i=1}^{n}\log\left(\int_{\Theta }f\left(y_{i}|\theta_{i}\right)\;d\pi \left(\theta_{i}\right)\right).$$

The symbol Π represents the set of all possible probability density functions, which are defined on the parameter space Θ. This optimization process does not rely on any specific parameterization. Therefore, it provides a high degree of model flexibility. Regardless of whether it is discrete or continuous, the solution $\hat{\pi }$ of (3) exists and is unique, which can ensure that the optimization problem has a solution under reasonable constraints. In addition, under finite sample conditions, the maximum likelihood estimation tends to construct a solution through finite support points to maximize the log-likelihood function. Although the true mixture density π may be a continuous distribution, the solution of $\hat{\pi }$ is almost a discrete distribution, and the number of data points it supports is at most *n*. It can better adapt to the log-likelihood optimization problem of finite sample data. Many numerical optimization algorithms can be used to solve the maximization problem, such as the EM algorithm [15,16] and variational inference [17,18].

When the underlying distribution is reasonably considered to be a continuous distribution, the discrete form of the estimator shows consistency and excellent optimality in theory. Its discrete nature may lead to a significant decrease in the estimation effect. Because the discrete solution only distributes the probability mass on a limited number of support points, it cannot effectively capture the smooth transition and local detail characteristics inherent in the continuous distribution.

Researchers have proposed related smoothing variants [19]. These variants attempt to make the estimated results closer to the true continuous distribution by introducing additional smoothing mechanisms. Techniques such as bandwidth adjustment [20], roughness penalty [21], and spline fitting [22] are used widely. However, these smoothing techniques usually require complex parameter adjustments, and the quality of parameter selection directly affects the accuracy and stability of the estimation. A small bandwidth may lead to oversmoothing and be unable to get rid of discrete characteristics, while a big bandwidth may cause oversmoothing. In addition, the parameter dependence and computational complexity between different methods also bring challenges to large-scale datasets.

In statistical inference and model estimation, bootstrapping has been shown to be a very effective tool for simulating continuous densities from prior and posterior distributions [23]. The bootstrap repeatedly samples the data to generate multiple subsets. It performs statistical inference on each subset. The goal is to generate a new dataset by introducing perturbations to the observed data. The repeated estimate π is based on these datasets, eventually converging to a more stable and smoother approximation of π. A natural approach is to replace resampling with random weights $w=\left(w_{1},w_{2},\ldots ,w_{n}\right)\in R^{n}$, forming a weighted bootstrap. The importance of each sample point $y_{i}$ in the bootstrap sample is determined by the weight $w_{i}$. The corresponding bootstrap version of the log-likelihood function is calculated by the following equation.

(4) $${\hat{\pi }}_{w}=\underset{\pi \in \Pi }{\arg\max}\sum_{i=1}^{n}w_{i}\cdot \log\left(\int_{\Theta }f\left(y_{i}|\theta_{i}\right)\;d\pi \left(\theta_{i}\right)\right).$$

By repeatedly sampling and estimating different weight vectors $w^{\left(1\right)},\ldots ,w^{\left(B\right)}$, we can obtain mixture density estimators ${\hat{\pi }}_{w^{\left(1\right)}},\ldots ,{\hat{\pi }}_{w^{\left(B\right)}}$, and smoothly integrate them into an approximation of a continuous mixture density.

(5) $$\bar{\pi }\left(\theta \right)=\frac{1}{B}\sum_{b=1}^{B}{\hat{\pi }}_{w^{\left(b\right)}}\left(\theta \right)$$

By introducing bootstrap weights, the choice of weight distribution has a significant impact on estimation performance and computational characteristics. Generally, there are two classic choices. One is to use the multinomial distribution, i.e., w∼Multinomial(n,(11nn,…,11nn)), which corresponds to the traditional nonparametric bootstrap. The other is to use the Dirichlet distribution, i.e., $w∼n\times Dirichlet(n,I_{n})$, which corresponds to the weighted likelihood bootstrap.

The weight $w_{i}$ is a non-negative integer representing the number of times the *i*-th observation is drawn in resampling with replacement. The total weight is strictly *n*. This approach is intuitive and completely consistent with the classic bootstrap. The weights are discrete integers; it is easy for some observations in a given sample to have zero weights. The discrete weights can cause the subsequent optimization objective function to be less smooth, thus affecting the optimization efficiency and stability of gradient-based methods.

By using a Dirichlet distribution to generate weights, $w_{i}$ is a continuous positive real number with $\sum_{i=1}^{n}w_{i}^{\left(b\right)}\approx n$. The weight of each sample is different, which improves the diversity and consistency of bootstrap samples. The continuous weights ensure smoother optimization of the weighted likelihood function, which facilitates stable training for deep learning methods such as neural network generators. We chose to use a Dirichlet distribution to generate weights. This choice not only enhances the training stability but also results in more efficient and scalable mixture distribution estimation in practical applications.

There always exits a cost issue in the calculation [24]. In the optimization process, it is often necessary to calculate high-dimensional integrals. When high dimensions exit, such integral problems cannot be solved analytically and must be approximated by numerical methods. If the data needs to be resampled several times and the sample size *n* is large, the computational and memory costs will increase exponentially. The bootstrap for large-scale datasets faces computational difficulty. Secondly, bootstrapping often introduces weight randomness to approximate the smooth distribution, meaning that the results may not be smooth enough [25]. According to the Kiefer–Wolfowitz theorem [26], the distribution estimated by bootstrap must be the number of support points at most *n*. If multiple resampling is performed, the final estimate generated will still be a discrete distribution [27]. The final result is a combination of multiple discrete distributions. Although this combination can approximate the true distribution in some cases, it usually cannot provide sufficient smoothness. In the high-dimensional data or complex distributions, this discrete estimation often manifests as local ‘spike’ characteristics and lacks the smooth transition of the true distribution, causing the model estimation to deviate from the continuity of the true distribution.

Latent mixture models and their semiparametric maximum likelihood estimators provide a flexible foundation for density estimation. Bootstrap-based variants further introduce smoothing effects and enhance robustness, yet they inevitably suffer from high computational costs and a lack of smoothness due to their discrete nature. These limitations highlight the need for an efficient and expressive approaches. In this work, we address these challenges by introducing a fast generative bootstrap framework. It retains the flexibility of MLE while overcoming the computational and smoothness issues.

### 2.2. Bayesian Networks and TAN Model

Bayesian networks (BNs) are probabilistic graphical models that combine probability theory with graph theory [28]. Their structure is represented by a directed acyclic graph (DAG), where nodes denote random variables and edges capture conditional dependencies. The key idea of BNs is to decompose the joint probability distribution according to the graphical structure. It enables efficient modeling and inference in high-dimensional scenarios. If the network consists of *d* nodes $X_{1},\ldots ,X_{d}$, the joint distribution can be factorized as the following equation.

(6) $$p\left(y_{1},\ldots ,y_{d}\right)=\prod_{i=1}^{d}p\left(y_{i}|\mathrm{Pa}\left(X_{i}\right)\right)$$

where $Pa(X_{i})$ denotes the parent set of node $X_{i}$. This factorization relies on conditional independence assumptions, which allow complex global distributions to be expressed by local conditional distributions. The learning of Bayesian networks typically involves two tasks, which are structure learning and parameter learning. Structure learning aims to determine the network, which reflects the dependency relations among variables, while parameter learning estimates the conditional probability distributions $p(y_{i}|Pa\left(Y_{i}\right))$ given the structure [29]. Its search space grows super-exponentially with the number of variables. To tackle this, researchers have proposed a range of methods, including score-based search, constraint-based independence tests, and hybrid approaches [30]. These methods balance expressive power and computational feasibility, making BNs widely applicable in practice.

Naive NB assumes conditional independence among features, which ensures high efficiency but may lead to performance degradation due to its unrealistic assumptions. To overcome this, the TAN model was proposed, which relaxes the independence assumption by allowing each feature. It depends not only on the class label *c* but also on another features, thus forming a maximum spanning tree structure. The class-conditional factorization is given by the following equation.

(7) $$p\left(y_{1},\ldots ,y_{d}|c\right)=\prod_{i=1}^{d}p\left(y_{i}|\mathrm{Pa}\left(X_{i}\right),c\right)$$

The critical challenge in TAN learning lies in selecting an appropriate tree structure [31]. A common strategy is to use CMI to measure the dependency between features.

(8) $$I\left(X_{i};X_{j}|C\right)=\sum_{c}p\left(c\right)\int p\left(y_{i},y_{j}|c\right)\log\frac{p(y_{i},y_{j}|c)}{p\left(y_{i}|c\right)\;p\left(y_{j}|c\right)}\;dy_{i}\;dy_{j}.$$

The above equation shows the calculation of CMI. The larger the CMI, the stronger the conditional dependency between two features, and thus the higher priority for establishing an edge in the TAN tree. A maximum spanning tree (MST) algorithm is applied to construct the dependency graph, which is then combined with the class node to form the complete TAN model [32]. This procedure enables TAN to retain the computational simplicity of NB while capturing essential feature dependencies. It improves predictive performance and interpretability. Nevertheless, the accuracy of TAN heavily relies on the reliability of CMI estimation, especially for continuous variables where poor density estimation can degrade structure. To address this issue, the proposed FGBMLE framework provides high-quality density estimates, ensuring robust and accurate TAN structure learning.

## 3. Structural Learning Process Based on the TAN-FGBMLE

### 3.1. FGBMLE Two-Stage Algorithm

**Figure 1:**
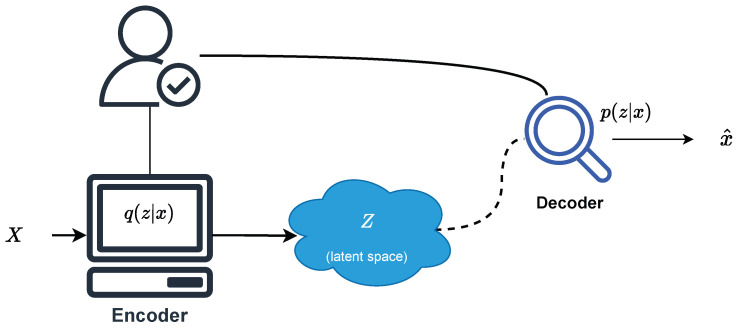
Graph network using autoencoder.

Generative learning has made significant progress, such as variational autoencoders (VAEs) [33] and generative adversarial networks (GANs) [34]. They have demonstrated powerful capabilities in unsupervised learning, image generation, and high-dimensional data modeling. As shown in Figure 1, VAEs learn data generation mechanisms in the latent variable space *z* by jointly training the encoder q(z|x) and the decoder p(x|z). Its optimization goal is to maximize the log-marginal likelihood logp(x) of the observed data, and to achieve this by optimizing the evidence lower bound.

(9) $$\log p\left(x\right)⩾E_{q(z|x)}\left[\log p\left(x|z\right)\right]-KL\left(q\left(z|x\right)||\;p\left(z\right)\right)$$

Here, p(z) is a pre-set prior distribution of the latent variable, typically a standard normal distribution. The encoder learns to encode the input sample *x* into a latent space representation *z*. The decoder reconstructs the sample $\hat{x}$ based on *z* and jointly optimizes the reconstruction error with the Kullback–Leibler (KL) divergence to capture the underlying structure of the data. By using the latent variable learning and sampling mechanism, VAEs can effectively approximate the complex distribution of observed data in the latent space.

This variational lower bound (ELBO) serves as a conceptual foundation for our proposed FGBMLE framework. While we do not directly optimize this bound, it inspires the design of our generator-based likelihood estimation: instead of explicitly computing the variational posterior q(z|x), the generator G(w,z) implicitly learns to produce latent representations θ that maximize the weighted likelihood under bootstrap perturbations. Hence, the ELBO highlights the connection between traditional variational inference and our implicit generative estimation approach.

Inspired by the general philosophy of deep generative models such as VAEs, we rethink the estimation of the latent mixture distribution π(θ). The central idea is to construct a neural mapping from an easy-to-sample distribution to a complex target distribution, without requiring an explicit parametric form for π(θ). This implicit generative formulation provides high flexibility and strong expressive power in high-dimensional latent spaces. A generator can learn structural patterns of the latent distribution directly from data-driven signals, thus enabling mixture modeling without explicit density assumptions.

In the traditional MLE framework, we typically estimate the latent distribution π(θ) by maximizing the log-likelihood function. This process relies on density estimation of the sample data, especially when multiple bootstrap sampling is involved. We introduce a parameterized neural network generator G(w,z), where *w* represents the bootstrap weights, *z* is random noise sampled from an underlying distribution, and the network outputs a latent variable θ=G(w,z), thus implementing a nonlinear generative process that maps the guided weights to the space of the mixture distribution. This method essentially attempts to mimic the sample generation behavior of the bootstrap under different weight configurations, but instead of solving the optimization problem separately each time, a unified estimation function is obtained through end-to-end training. For a given weight *w*, we hope that the latent variable output by the generator has as high a weighted marginal likelihood as possible on the observed data. Due to the randomness of the generator output, it is necessary to average the noise *z* [35] to obtain the expected likelihood of the observed samples under the latent variable generation. In order to reflect the overall performance under different weight perturbations, it is also necessary to take the expectation on the weight *w*. Therefore, the training objective of the generator can be formalized as the following equation.

(10) $$\hat{G}=\arg\max_{G}\;E_{w}\left[\sum_{i=1}^{n}w_{i}\cdot \log\left(E_{z}\left[f(y_{i}|G\left(w,z\right))\right]\right)\right]$$

The expectation comes from the bootstrap weights *w*, while the inner expectation represents the likelihood of the generator’s output samples under the observation model $f(y_{i}|\theta_{i})$ by given the weights. In this objective function, the inner expectation $E_{z}$ is responsible for integrating the uncertainty in latent variable generation caused by noise, ensuring that the generator’s output under different noise sampling conditions. It has good average likelihood performance for the observed data. The outer expectation $E_{w}$ simulates the generator’s robustness on the entire dataset under different bootstrap sampling weights. By jointly optimizing these two layers of expectations, the generator *G* can learn a method to quickly generate latent parameter samples.It avoids the computational overhead of resolving the problem for each bootstrap in traditional methods.

Although the initial training phase of the generator introduces a higher computational load due to the end-to-end optimization process, this cost is incurred only once. Once trained, the generator can directly produce latent samples θ=G(w,z) without solving separate optimization problems for each bootstrap weight configuration, resulting in a substantial reduction in inference time. In practice, the initialization stage typically takes about 1.2–1.5 times the cost of a single bootstrap optimization run, but subsequent inference is accelerated by more than an order of magnitude. Thus, the overall computational trade-off favors the FGBMLE approach, as the amortized inference efficiency significantly outweighs the one-time training overhead.

With this strategy, we successfully transform the repeated optimization bootstrap process into a one-time generator training, significantly reducing the overall computational burden. We use a feedforward neural network (FNN) to construct *G* [36]. FNNs have universal approximation properties for a large number of functions [37]. Each layer of the neural network can automatically learn important features and patterns in the data. When the network is used as a generator, the network itself does not rely on any assumptions and it can approximately approach the true distribution of the data. In order to ensure that the generator receives different noise input *z* at each iteration, we sample z∼Unif(0,1) from a uniform distribution. The bootstrap weight *w* is the importance assigned to each sample during the training process. In some datasets, the distribution of samples may be unbalanced, so we sample from a Dirichlet distribution, i.e., $w∼n\times Dirichlet(n,I_{n})$, where $I_{n}$ is an *n*-dimensional all-ones vector producing normalized non-negative weights $w_{1},\ldots ,w_{n}$. This imbalance is solved by appropriate weighting to prevent the model from focusing only on certain samples during training [38].

Although the generator alone can already approximate the target density, the subsequent candidate generation and two-stage optimization further refine both the component weights and the mixture structure. Empirically, we observed that using only the generator (without Stages I and II) results in a relatively small improvement in the average negative log-likelihood (NLL), typically less than 1–2% after convergence. In contrast, when the candidate generation and bootstrap refinement stages are included, the improvement in NLL usually reaches 10–15% for one-dimensional data and around 8–12% for two-dimensional cases. Furthermore, the final model produces noticeably smoother estimated densities and exhibits better generalization on unseen samples. Therefore, although the initial generator provides a strong baseline, the two-stage refinement significantly enhances both the accuracy and stability of the overall density estimation process.

The latent variables θ in such models often exist in spaces with tens or even hundreds of dimensions d≫1. While this high-dimensional representation can capture complex data structures such as multimodal distributions and nonlinear dependencies, it also imposes a significant computational burden and makes statistical inference difficult [39].

Traditional maximum likelihood estimation methods encounter significant bottlenecks in this area. As the dimension increases, the computational complexity of the optimization process increases exponentially, making standard algorithms impractical for d>10. When using bootstrap methods to quantify uncertainty, each resampling requires resolving the high-dimensional optimization problem, quickly becoming computationally prohibitive. This “curse of dimensionality” severely restricts the practical application of high-dimensional latent variable models.

To improve the model’s expressiveness and training stability for high-dimensional nonparametric mixture densities, we expand the output of the generator G(w,z) from a single parameter vector $\theta \in R^{d}$ to a sequence of candidate variables $\theta ={\left\{\theta^{\left(j\right)}\right\}}_{j=1}^{l}$, where each $\theta^{\left(j\right)}\in R^{d}$ represents a set of selectable mixture parameter vectors. This eliminates the generator output as a single point estimate and instead creates a high-dimensional support set, allowing it to approximate more complex density structures. The primary purpose of this approach is to effectively reduce the variance of Monte Carlo expectation estimate by introducing multiple candidate samples, thereby improving the stability of the gradient estimate in stochastic gradient descent [40].

**Figure 2:**
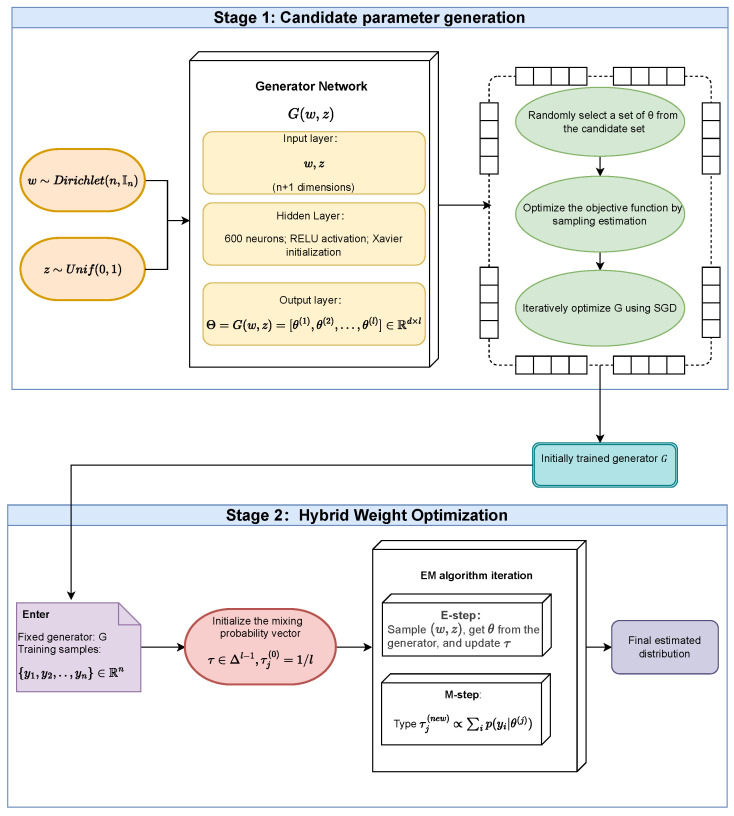
Two-stage algorithm framework of FGBMLE.

There are often statistical correlations between multiple generated samples, and directly averaging them may lead to estimation bias. We further introduce a mixed probability vector $\tau =(\tau_{1},\ldots ,\tau_{l})$ to perform weighted sampling on each $\theta^{\left(j\right)}$ in the candidate set. It obtains independent bootstrap samples while maintaining expressiveness. At each sampling, an item τ is randomly selected from j=1,…,l according to the $\theta^{\left(j\right)}$ distribution. This process is repeated several times. To achieve this process, we design the following two-stage training framework in Figure 2.

Given a dataset $Y={\left\{y_{i}\right\}}_{i=1}^{n}$, the Stage-I training introduces two types of random variables as input to the generator. One is the bootstrap weight vector $w∼n\times Dirichletn,I_{n}$ sampled from the Dirichlet distribution. The other is the potential noise z∼Unif(0,1) that follows a uniform distribution. After receiving these two inputs, the generator G(w,z) outputs a candidate parameter matrix.

(11) $$\Theta =G\left(w,z\right)=\left[\theta^{\left(1\right)},\theta^{\left(2\right)},\ldots ,\theta^{\left(l\right)}\right]\in R^{d\times l}$$

The each column $\theta^{\left(j\right)}\in R^{d}$ is a candidate density parameter. *l* represents the number of candidates output by the generator. This allows the generator to simultaneously provide multiple candidate solutions in different regions of the latent distribution, providing richer structure for subsequent modeling. Initially, τ is set to a uniform distribution $\tau_{0}=\left(1/l,\ldots ,1/l\right)$. Subsequently, during training, an index variable *r* is extracted via $r∼Multinomial(1,\tau_{0})$ and indicates the selected candidate parameter in a one-hot fashion. At each iteration, *r* randomly selects a parameter $\theta^{\left(r\right)}$ from the candidate set $\left\{\theta^{\left(1\right)},\ldots ,\theta^{\left(l\right)}\right\}$ and uses it in the likelihood calculation. This random sampling strategy allows the model to traverse all candidates in the desired sense, but a single update depends on only one of them, thus avoiding the dilution effect caused by averaging. So the optimization goal of Stage-I is defined as the following equation.

(12) $$L_{1}\left(G\right)=-E_{w,z,r}\left[\sum_{i=1}^{n}w_{i}\cdot \log f\left(y_{i}|\theta^{\left(r\right)}\right)\right],r∼Multinomial\left(1,\tau_{0}\right)$$

The $f(y_{i}|\theta^{\left(r\right)})$ represents the probability density of sample $y_{i}$ under the parameter $\theta^{\left(r\right)}$. This objective combines bootstrap weights and candidate sampling to effectively control the estimation variance while ensuring that the generator covers a wide range of underlying distribution structures. Theoretically, it satisfies the following inequality.

(13) $$Var_{z}\left[f\left(y_{i}|\theta_{i}\right)\right]⩾Var_{z}\left[E_{r}f\left(y_{i}|\theta^{\left(r\right)}\right)\right]$$

By introducing candidate structures and random sampling, the variance of the training process can be effectively suppressed, thereby improving the stability of the estimation.

During the optimization process, we use stochastic gradient descent (SGD) to update the generator parameter θ and continuously adjust the expressive power of the candidate structure. Finally, the optimization result of Stage-I can be expressed as the following equation.

(14) $$G^{*}=\underset{G}{\arg\max L_{1}\left(G\right)}$$

Here, $G^{*}$ represents the generator that achieves the optimality under the objective function $L_{1}\left(G\right)$. This stage of training lays the initial foundation for subsequent more sophisticated modeling in Algorithm 1.
**Algorithm 1** FGBMLE Stage-I**Input:** Dataset $Y={\left\{y_{i}\right\}}_{i=1}^{n}$; epochs *T*; candidate number *l*; initial generator $G(\cdot ;\theta_{0})$; uniform prior $\tau_{0}=\left(1/l,\ldots ,1/l\right)$; learning rate η. **Output:** generator $G^{*}$ producing candidate set $\left\{\theta^{\left(1\right)},\ldots ,\theta^{\left(l\right)}\right\}$.  1: **for** t=1 to *T* **do**  2:       Sample bootstrap weights: $w∼Dirichlet(n,I_{n})$  3:       Sample latent noise: z∼Unif(0,1)  4:       Generate candidate parameters: $\Theta =G\left(w,z\right)=\left[\theta^{\left(1\right)},\ldots ,\theta^{\left(l\right)}\right]$  5:       Sample index variable: $r∼Multinomial(1,\tau_{0})$  6:       Compute objective $L_{1}\left(G\right)$ using Equation (12)  7:       Update generator parameters θ via SGD using learning rate η  8: **end for**  9: **return** 
$G^{*}$

For all experiments, the proposed FGBMLE model was implemented using a fully connected generator network with three layers (dimensions 256–128–*d*) and ReLU activations. The model was optimized with the Adam optimizer at a fixed learning rate of $1\times 10^{-3}$ across all tasks, ensuring comparable convergence behavior. Training was performed with a batch size of 128 and Xavier initialization for all weights. Early stopping was applied when the validation negative log-likelihood (NLL) improvement dropped below $10^{-4}$ for ten consecutive epochs, effectively preventing overfitting. The number of training epochs *T* was set to 500 for one-dimensional data, 800 for two-dimensional data, and 1000 for the TAN experiments. The number of candidate components *l* was fixed at 50 in most cases, and adjusted within the range of 20–80 depending on data complexity to balance expressiveness and computational cost.

In Stage-II, we improve the density estimate by optimizing the mixture weights associated with the set of candidate parameters generated in the first stage. We introduce a mixture weight vector $\tau =(\tau_{1},\ldots ,\tau_{l})$, which belongs to the *l*-dimensional probability vector, i.e., $\tau_{j}\geq 0$ and $\sum_{j=1}^{l}\tau_{j}=1$. This weight vector is used to combine the candidate parameters into a mixture model. At this stage, the estimated density of data sample $y_{i}$ can be expressed as the following equation.

(15) $$\hat{p}\left(y_{i}\right)=\sum_{j=1}^{l}\tau_{j}\cdot E_{w,z}\left[f(y_{i}|\theta^{\left(j\right)})\right]$$

Here, $G^{*}$ is the generator trained in Stage-I, $\theta^{\left(j\right)}$ represents the *j*th candidate parameter generated by $G^{*}\left(w,z\right)$. The $E_{w,z}\left[\cdot \right]$ represents the expectation over the guided weights $w∼Dirichlet(n,1_{n})$ and the underlying noise z∼Unif(0,1). This formula allows the model to aggregate information from multiple candidate parameters. It also retains flexibility in representation.

To determine the optimal mixture weights, we minimize the negative log-likelihood.

(16) $$L_{2}\left(\tau \right)=-\sum_{i=1}^{n}\log\left(\sum_{j=1}^{l}\tau_{j}\cdot E_{w,z}\left[f\left(y_{i}|\theta^{\left(j\right)}\right)\right]\right)$$

It makes the mixture weights more consistent with the empirical distribution of the dataset. The τ optimization is performed by using the Monte Carlo Expectation Maximization (MCEM) algorithm [41]. In each iteration *t*, the update rule for the *j*th component is achieved by using the following equation.

(17) $$\tau_{j}^{\left(t+1\right)}=\frac{1}{n}\sum_{i=1}^{n}\frac{\tau_{j}^{\left(t\right)}\cdot E_{w,z}\left[f\left(y_{i}|\theta^{\left(j\right)}\right)\right]}{\sum_{j=1}^{l}\tau_{j}^{\left(t\right)}\cdot E_{w,z}\left[f\left(y_{i}|\theta^{\left(j\right)}\right)\right]}$$

The denominator ensures that the updated weights remain regularized on the simplex. The iterative process continues until it reaches convergence, which is determined by the tolerance criterion $∥\tau^{new}-\tau^{old}∥$.

This two-stage approach ensures that the generator provides diverse candidate structures in high-dimensional latent spaces, while the mixture weights refine the density estimation through adaptive reweighting. The combination of these two steps enables the model to capture complex high-dimensional distributions with improved accuracy and stability in Algorithm 2.
**Algorithm 2** FGBMLE Stage-II**Input:** Trained generator $G^{*}$; dataset $Y={\left\{y_{i}\right\}}_{i=1}^{n}$; tolerance tol; number of candidates *l*. **Output:** Optimized mixture weights $\tau^{new}=\left(\tau_{1},\ldots ,\tau_{l}\right)$.  1: Initialize: $\tau^{new}=\left(1/l,\ldots ,1/l\right)$, $\tau^{old}=\left(0,\ldots ,0\right)$  2: **while**$\min_{j}\left|\tau_{j}^{old}-\tau_{j}^{new}\right|\geq tol$ 
**do**  3:      Set $\tau^{old}=\tau^{new}$  4:      **for** j=1 to *l* **do**  5:            Sample bootstrap weights: $w∼Dirichlet(n,I_{n})$  6:            Sample latent noise: z∼Unif(0,1)  7:            Update $\tau_{j}^{new}$ using Equation (17)  8:      **end for**  9: **end while** 10: **return** 
$\tau^{new}$

### 3.2. TAN-FGBMLE Framework

It is worth noting that the proposed FGBMLE framework is not limited to TAN models. The two-stage mechanism described above is a general estimation scheme that can be embedded into various probabilistic models, mixture estimators, and graph structures as long as the likelihood function f(y∣θ) is available. In this section, we instantiate FGBMLE using the TAN model as an example. TAN was chosen because its structure learning process heavily relies on accurate estimates of class-conditional margin densities and joint densities, making it an ideal testbed to demonstrate how FGBMLE improves density estimation and conditional dependency inference [42].

With the two-stage estimation method, this section applies it to TAN structure learning. The core idea is to leverage the rich candidate parameter space provided by the generator, achieve robust distribution estimates through flexible weighting, and then embed these estimations into the measure of conditional dependencies between features for TAN structure learning.

The marginal and joint distributions of features need to be calculated for each class *c*. Our random variables are denoted by capital letters $X_{i}$. Therefore, the data sample $y=(y_{1},\ldots ,y_{d})$ is considered to be a realization of the random vector $X=(X_{1},\ldots ,X_{d})$.

Directly adopting empirical frequency or kernel density methods usually leads to bias in small sample sizes, resulting in unstable mutual information estimation. We extend the two-stage FGBMLE estimator to the class-conditional scenario and give the following distribution estimation.

(18) $$\hat{p}\left(y_{i}|c\right)=\sum_{j=1}^{l}\tau_{i,c,j}E_{w,z}\left[f(y_{i}|\theta_{i,c}^{\left(j\right)})\right]$$

Here, $\theta_{i,c}^{\left(j\right)}$ represents candidate parameters generated by the Stage-I generator $G^{*}\left(w,z\right)$; $\tau_{i,c,j}$, which denotes the mixture weights optimized in Stage-II. The subscript i,c,j indicates that the weight corresponds to the *i*th feature, the category *c*, and the *j*th of the *l*th candidate parameters. The $E_{w,z}\left[\cdot \right]$ denotes the expectation with respect to bootstrap weights $w∼Dirichlet(n,I_{n})$ and latent noise z∼Unif(0,1). This design ensures that the estimated probability distributions retain smoothness and generalization ability even under finite samples.

After obtaining the class-conditional probability estimates, we can further define the conditional mutual information, which measures the dependency strength between features $X_{i}$ and $X_{j}$ given the class *c*.

(19) $$I\left(X_{i},X_{j}|C\right)=\sum_{c}\hat{p}\left(c\right)\;\int \int \;\hat{p}\left(y_{i},y_{j}|c\right)\log\frac{\hat{p}\left(y_{i},y_{j}|c\right)}{\hat{p}\left(y_{i}|c\right)\hat{p}\left(y_{j}|c\right)}dy_{i}dy_{j}$$

In this equation, $\hat{p}\left(c\right)$ denotes the empirical prior probability of class *c*. The denominator term $\hat{p}\left(y_{i}|c\right)\hat{p}\left(y_{j}|c\right)$ represents the product of the marginal distributions. The integral $\;\int \int \;dy_{i}dy_{j}$ is taken over the feature space of $\left(y_{i},y_{j}\right)$. The integral can be approximated by sampling from the estimated distribution and applying the Monte Carlo method. Compared to traditional mutual information computation, the FGBMLE smoothing terms in Equations (18) and (19) effectively reduce the bias caused by noisy samples, which will lead to more robust edge weights and improved reliability in structure learning.

Based on the values of conditional mutual information, a weighted complete graph can be constructed, and the maximum spanning tree algorithm can be used to extract the main dependencies among features. By orienting the spanning tree and adding the class node *c*, the complete TAN structure can be obtained. The conditional probability of each feature node given its parent node and class can be expressed as the following equation.

(20) $$\hat{p}\left(y_{i}|y_{pa},c\right)=\frac{\hat{p}\left(y_{i},y_{pa}|c\right)}{\hat{p}\left(y_{pa}|c\right)},\;\hat{p}\left(y|c\right)=\prod_{i=1}^{d}\hat{p}\left(y_{i}|c,Pa\left(X_{i}\right)\right)$$

Here, $y_{pa}$ denotes the observed value of the parent node of $X_{i}$ in the TAN structure, and $Pa(X_{i})$ denotes the parent set of feature $X_{i}$. By combining with the class prior $\hat{p}\left(c\right)$, the classification rule can be written as the following equation.

(21) $$\hat{c}\left(y\right)=\arg\max_{c}\;\hat{p}\left(c\right)\hat{p}\left(y|c\right)$$

This modeling approach remains consistent with the original definition of TAN, but incorporates the two-stage mechanism of FGBMLE into the estimation process, significantly enhancing the stability of mutual information calculation and the accuracy of edge structure learning [43].

Since the structure of TAN relies on the precise calculation of class-conditional mutual information. With estimated the class-conditional mutual information matrix between continuous attributes using the FGBMLE method, and used this as the edge weights of the graph. The class-conditional mutual information is calculated according to Formula (8), where $y_{i}$ and $y_{j}$ represent pollutants or environmental factors, and *C* denotes the air quality level. This metric quantifies the dependency strength between two variables under given classification conditions.

The Prim algorithm was applied to construct a maximum spanning tree. It obtains the optimal TAN dependency structure. The basic process of Prim’s algorithm is to start from any node and iteratively add the edge with the maximum weight that connects a new node to the existing tree, until all nodes are included. Mathematically, this can be formalized as the following:

(22) $$T^{*}=\underset{T\subseteq G}{\arg\max}\sum_{(Y_{i},Y_{j})\in T}I_{c}\left(Y_{i};Y_{j}\right),$$

where $I_{c}\left(Y_{i};Y_{j}\right)$ represents the class-conditional mutual information and $T^{*}$ is the resulting optimal dependency tree.

Overall, this integration shows how the general FGBMLE estimator can serve as a plug-and-play density estimation engine within the TAN framework in Algorithm 3. While the present work focuses on TAN as a demonstrative application, the same mechanism can be naturally extended to more general Bayesian network structures, mixture-based models, or continuous graphical learning tasks where accurate conditional density estimation is required. This highlights that FGBMLE is a broadly applicable framework, with TAN structure learning being only one of its practical instantiations.
**Algorithm 3** TAN-FGBMLE**Input:** Dataset $Y={\left\{y_{i}\right\}}_{i=1}^{n}$; trained generator $G^{*}$ from Algorithm 1; optimized mixture weights τ from Algorithm 2; number of features *d*; number of candidate parameters *l*. **Output:** TAN structure and classification rule $\hat{c}\left(y\right)$.  1: **for** each class *c* **do**  2:       Estimate marginal distributions $\hat{p}\left(y_{i}|c\right)$ for all i=1,…,d using Equation (18)  3:       Compute conditional mutual information $I(X_{i},X_{j}|C)$ using Equation (19)  4: **end for**  5: Construct a weighted complete graph with edge weights $I(X_{i},X_{j}|C)$  6: Extract maximum spanning tree to determine feature dependencies  7: Orient the spanning tree and add class node *c* to obtain TAN structure  8: **for** each feature $X_{i}$ **do**  9:       Compute conditional probability $\hat{p}\left(y_{i}|y_{pa},c\right)$ using Equation (20) 10: **end for** 11: Define classification rule $\hat{c}\left(y\right)=\arg\max_{c}\hat{p}\left(c\right)\hat{p}\left(y|c\right)$ 12: **return** TAN structure and $\hat{c}\left(y\right)$

## 4. Experiment Results

### 4.1. Simulation Experiments

In order to systematically evaluate the performance of TAN-FGBMLE, we designed a series of simulation experiments. These experiments cover typical potential distribution characteristics. They aim to test the ability of our method to capture complex distribution structures, handle different sample sizes, and achieve efficient computation. Specifically, we define two simulation scenarios with Table 1, presenting bimodal and unimodal restricted distributions.

In the GMM scenario, the latent distribution has a bimodal structure, which is suitable for testing the performance. In the GaMM scenario, the latent variables are restricted to the high-dimensional space $[0,1{]}^{d}$. This spatially restricted latent distribution is used to evaluate the adaptability of our method to constrained unimodal distributions. We set the data size to *n* = 1000 and further expanded it to *n* = 10,000 and *n* = 100,000 to evaluate computational efficiency. The generator structure of FGBMLE uses a two-layer fully connected neural network, each containing 600 neurons. ReLU is used as the activation function, and the parameters are initialized using Xavier, which is a commonly used weight initialization function designed to avoid gradient vanishing or exploding problems. Training is performed using the Adam optimizer, with an initial learning rate of 0.001. Bootstrap weights *w* are generated using a Dirichlet distribution. All experiments are based on the PyTorch 2.2.2 framework and run on an NVIDIA GeForce RTX 4060 Ti GPU to ensure efficient computational performance.

**Figure 3:**
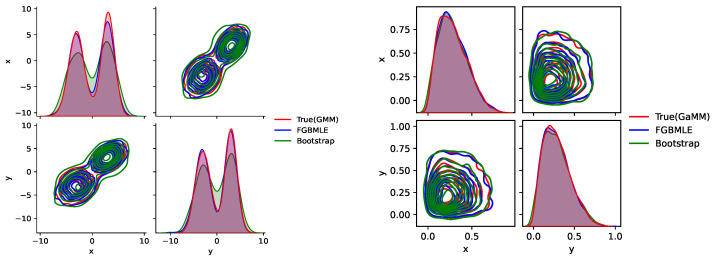
Estimating the probability density using two-dimensional data.

Figure 3 comprehensively demonstrates the ability of FGBMLE and bootstrap methods to fit the underlying distribution π(θ) in two typical simulation scenarios, corresponding to GMM and GaMM, respectively. Figure 3 shows that in the GMM scenario, the true distribution exhibits two distinct peaks, located near θ = −3 and θ = 3, respectively. FGBMLE almost perfectly reproduces the height and position of these two peaks, demonstrating its strong ability to capture multimodal distributions. Bootstrap also performs well, but its fit to the peak height is slightly insufficient. In the GaMM scenario, the true distribution is unimodal, located in the interval (0, 1). FGBMLE accurately captures this unimodal characteristic, and its smooth curve is very close to the true distribution, especially in terms of the peak position and distribution shape. Bootstrap has slight deviations in fitting unimodal characteristics, especially near the boundaries.

**Figure 4:**
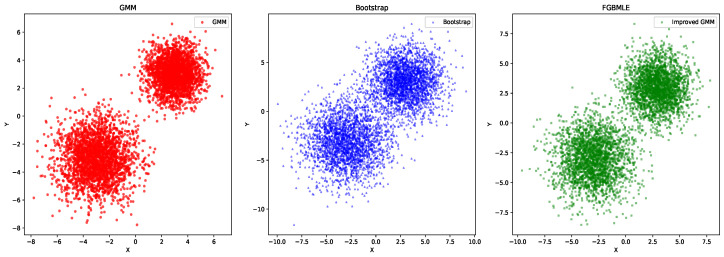
Sampling performance comparison using GMM.

The results in Figure 4 clearly show that FGBMLE exhibits excellent fitting capabilities in multimodal and unimodal restricted scenarios. The smooth distribution it generates can not only accurately capture the detailed characteristics of the real distribution, but also avoid the shortcomings of traditional methods caused by oversmoothing, verifying its superiority in complex distribution modeling.

Table 2 compares the performance of FGBMLE, bootstrap, and KDE using four complementary evaluation metrics. The 1-Wasserstein distance, $W_{1}\left(\pi ,\hat{\pi }\right)=\int \left|F_{\pi }\left(x\right)-F_{\hat{\pi }}\left(x\right)\right|\;dx$, where *F* denotes the cumulative distribution function, reflects global distributional discrepancies. The integrated squared error (ISE), $ISE=\int_{R^{d}}{\left\{\hat{\pi }\left(x\right)-\pi \left(x\right)\right\}}^{2}\;dx$, measures local density fidelity. In addition, we report the mean squared error (MSE) between the estimated and true density values, as well as the Kullback–Leibler (KL) [44] divergence, which evaluates the probabilistic discrepancy between π and $\hat{\pi }$. Table 2 summarizes the averages over 50 independent simulation runs for both scenarios. The results show that FGBMLE performs comparable to or better than traditional bootstrap methods across all metrics, particularly in the GaMM scenario, while the KDE baseline serves as a nonparametric reference for comparison.

**Figure 5:**
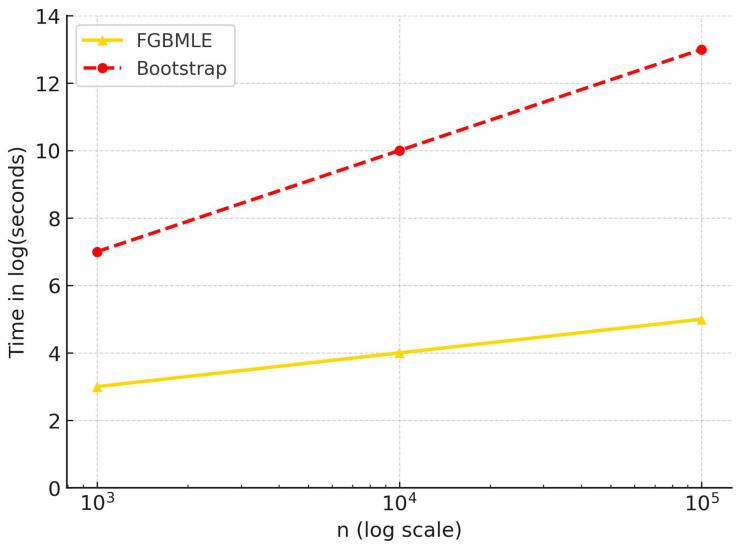
Average computation time for the different sample sizes.

We also compared the average computation time of FGBMLE and bootstrap in simulations with sample sizes n∈{1000,10,000,100,000}. The results are plotted on a logarithmic scale in Figure 5. This figure shows that FGBMLE is more scalable. Specifically, when *n* = 100,000, FGBMLE completes in only approximately 5 min, while bootstrap takes nearly triple the amount of time.

To validate the applicability of the proposed TAN-FGBMLE method in complex distribution scenarios, we designed a probability density estimation experiment based on artificial data. The experimental data consists of three Gaussian components, $N(-3,0.5^{2})$, $N(0,1^{2})$, and $N(3,0.7^{2})$, forming a typical trimodal, long-tailed, and heteroskedastic distribution. We compared the proposed method with traditional KDE and GMM, using real distributions as a benchmark.

To keep the evaluation framework consistent with Table 2, we also assess KDE, GMM, and TAN-FGBMLE under the trimodal long-tailed distribution using the same four quantitative metrics: W1, ISE, MSE, and KL divergence.

Table 3 reports the quantitative density estimation results for the trimodal distribution experiment using four evaluation metrics. As shown in the table, TAN-FGBMLE achieves the lowest values across all four metrics, indicating that its estimated density is consistently closer to the true distribution than those produced by KDE and GMM. In particular, the improvements in W1 and KL suggest that TAN-FGBMLE captures both the global distributional structure and the probabilistic discrepancies more effectively. The reduction in ISE and MSE further demonstrates its accuracy in recovering the local shape of the trimodal density. Overall, these results highlight the advantage of TAN-FGBMLE in modeling complex multimodal distributions.

**Figure 6:**
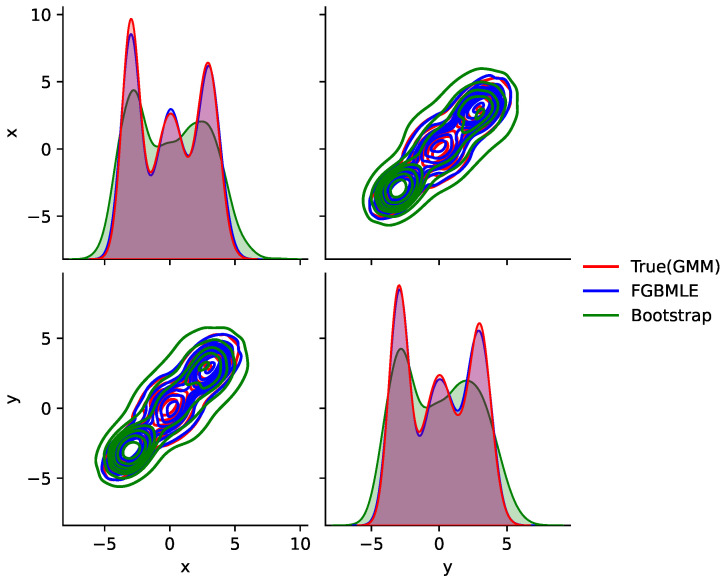
Density estimation results of the proposed TAN-FGBMLE method under a 2D trimodal distribution.

Figure 6 compares the density estimation results of the proposed FGBMLE with the true GMM and bootstrap NPMLE under a 2D trimodal distribution. FGBMLE reconstructs the three modal regions accurately and maintains sharp, well-aligned contour structures. Conversely, the bootstrap estimator produces overly dispersed contours and fails to preserve the multimodal shape. The results show that FGBMLE achieves clearly superior performance in capturing multimodal and heteroskedastic density structures in higher-dimensional settings.

### 4.2. Structure Recovery Experiment

To further evaluate the accuracy of the learned structures, a simulation-based experiment was conducted using data generated from a known continuous Tree-Augmented Naive Bayes model. In this model, one binary class variable C∈{0,1} and eight continuous attributes $X_{1},X_{2},\ldots ,X_{8}$ were considered. The underlying TAN structure over the attributes was predefined as a fixed chain, for example, $X_{1}\to X_{2}\to \ldots \to X_{8}$, and remained constant throughout all trials.

For each class *c*, the joint distribution $P(X_{1},\ldots ,X_{8}|C=c)$ followed a linear Gaussian model, where the root node $X_{1}$ was generated according to

(23) $$X_{1}|C=c∼N\left(\mu_{1}^{\left(c\right)},\sigma_{1}^{2}\right),$$

and each non-root node $X_{j}$ was generated from its attribute parent pa(j) as

(24) $$X_{j}|X_{\mathrm{pa}(j)},C=c∼N\left(a_{j}^{\left(c\right)}X_{\mathrm{pa}(j)}+b_{j}^{\left(c\right)},\sigma_{j}^{2}\right),$$

where all parameters were randomly initialized and fixed across experiments to ensure reproducibility. The class prior was set to P(C=1)=0.5.

Two synthetic datasets were generated with sample sizes n=1000 and n=2000, and each configuration was repeated for twenty independent trials. For each dataset, three methods, TAN-KDE, TAN-GMM, and the proposed TAN-FGBMLE were used to estimate the TAN structure by calculating conditional mutual information between attributes given the class variable and constructing a maximum-weight spanning tree. In all methods, the structure learning process was identical except for the underlying conditional density estimator used for CMI computation.

To measure the accuracy of the recovered structures, we adopted the Structural Hamming Distance (SHD) [45] between the estimated and the true attribute tree. Both trees were represented as undirected adjacency matrices $A^{\mathrm{true}}$ and $A^{\mathrm{est}}$ of dimensions d×d, and the SHD was defined as

(25) $$\mathrm{SHD}\left(A^{\mathrm{true}},A^{\mathrm{est}}\right)=\sum_{1\leq i<j\leq d}I\left[A_{ij}^{\mathrm{true}}\neq A_{ij}^{\mathrm{est}}\right],$$

where I[·] denotes the indicator function. SHD quantifies the number of edge additions or deletions required to transform the estimated tree into the true one. Only attribute–attribute edges were considered, excluding those connected to the class node *C*. For each method and sample size, we report the mean and standard deviation of SHD values averaged over twenty repetitions.

As shown in Table 4, the SHD values decrease with the increase in sample size, indicating that all methods benefit from larger datasets in structural learning. Among the three methods, FGBMLE-TAN consistently achieves competitive or superior SHD performance compared with KDE-TAN and GMM-TAN. In particular, when n=2000, FGBMLE-TAN obtains the smallest SHD value (0.2 ± 0.2), suggesting that the proposed method more accurately recovers the underlying TAN structure with sufficient data.

Although the SHD of TAN-FGBMLE is only marginally lower than that of TAN-GMM, it demonstrates higher stability (lower variance) across repeated trials. This result implies that the FGBMLE-based conditional density estimation provides a smoother and more robust approximation of the joint distribution, which effectively improves the reliability of structure learning. Consequently, the proposed TAN-FGBMLE achieves a better balance between structural accuracy and distributional modeling capability.

### 4.3. Comparative Experiments with Extended Naive Bayes and Discriminative Models

To further evaluate the effectiveness of the proposed TAN-FGBMLE model, we conducted comparative experiments against several state-of-the-art Naive Bayes extensions that explicitly model attribute dependencies, as well as strong discriminative classifiers. The compared Naive Bayes-type models include TAN, Averaged One-Dependence Estimators (AODEs) [46], Weighted AODE (WAODE) [47], Hidden Naive Bayes (HNB) [48], and Correlation-based Feature-Weighted Naïve Bayes (CFWNB) [49]. These models were selected because they relax the independence assumption of traditional NB by incorporating single or multiple parent dependencies among features. All implementations of the baseline algorithms are available in the WEKA platform, ensuring fair and reproducible comparisons.

In addition, three discriminative models, Logistic Regression, Random Forest (RF), and RBF-SVM, were included to assess whether the generative optimization of TAN-FGBMLE generalizes beyond the Naïve Bayes family. All models were trained under identical data partitions and preprocessing procedures. Two primary metrics were used for evaluation: classification accuracy (%) and average log-likelihood (LL). Each result represents the mean and standard deviation over five independent runs.

As shown in Table 5, TAN-FGBMLE consistently achieves superior results in both classification accuracy and log-likelihood compared with all competing models. Relative to the standard TAN, FGBMLE-TAN improves accuracy by 4.3% and significantly increases log-likelihood, indicating that the proposed generative Bayesian optimization effectively captures the conditional dependencies among features while maintaining a coherent probabilistic structure.

To assess the robustness of these improvements, we performed a Wilcoxon signed-rank test [50] across all datasets for pairwise comparisons between TAN-FGBMLE and each baseline model. The test statistic is defined as

(26) $$W=\min\left(W^{+},W^{-}\right),\;W^{+}=\sum_{d_{i}>0}R_{i},\;W^{-}=\sum_{d_{i}<0}R_{i},$$

where $d_{i}=x_{i}-y_{i}$ denotes the performance difference on the *i*-th dataset and $R_{i}$ is the rank of $|d_{i}|$. Under the null hypothesis that there is no systematic difference between models, FGBMLE-TAN was found to be significantly better than all other NB-type baselines at the 5% significance level (p<0.05) in Table 5.

Although discriminative models such as Random Forest and RBF-SVM also achieve competitive accuracy, TAN-FGBMLE slightly outperforms them in both metrics. This demonstrates that a well-trained generative structure can rival, and even surpass, discriminative models when handling complex or partially observed distributions, while also maintaining the interpretability advantages inherent to probabilistic graphical models.

### 4.4. Classification Performance on UCI Benchmark Datasets

To further evaluate the effectiveness of the proposed TAN-FGBMLE method on real-world classification tasks, we conducted experiments on 20 widely used UCI benchmark datasets. These datasets cover a wide range of scales, feature dimensions, and class complexity, providing a comprehensive testbed beyond controlled simulation environments. When encountering the issue of absent data points in the dataset, we implement a method of substituting them with the average value of each feature. The specific descriptive information of the dataset is shown in Table 6. We compared classification performance by comparing multiple classifiers, including Tree-Augmented Kernel Density Bayes (TAN-KDE), Naive Bayes classifier (NBC), Flexible Bayes Classifier (FBC), k-Nearest Neighbors (KNN), Decision Tree C4.5, Neural Network (NN), and Support Vector Machine (SVM). We used ten-fold cross-validation. The average classification accuracy is shown in Table 7.

From the Table 7, we can observe that there are clear differences in classification performance across different classifiers. For most datasets, TAN-FGBMLE achieves superior results compared to the baselines, demonstrating its ability to capture complex dependency structures more effectively. The SVM classifier chooses the RBF kernel, and its parameter adopts the lattice search method, c∈[−8,8], g∈[−8,8], with a step size of 0.1. Decision Tree uses the C4.5 model with post-pruning. The neural network parameters are set as $hidden_{l}ayer_{s}izes=100$, activation=relu, learning rate α= 0.001, $random_{s}tate=$ 42 and the max iteration $max_{i}ter=$ 1000. The nearest neighbor size is 5. The smoothing parameter for Gaussian is 0.001.

By using the 10-fold cross-validation, we obtain the mean and the variance of the classification accuracy. When the sample size is small or the feature dimension is limited, traditional classifiers such as NBC or SVM can still maintain reasonable accuracy due to their simple assumptions. However, as the number of samples and attributes increases, the advantage of TAN-FGBMLE becomes more evident, as it stabilizes mutual information estimation and reduces noise in high-dimensional spaces. Overall, TAN-FGBMLE improves average classification accuracy over TAN-KDE, NBC, FBC, KNN, C4.5, NN, and SVM by 3.1%, 2.4%, 3.6%, 2.7%, 4.2%, 1.5%, and 1.2%, respectively. These results confirm that TAN-FGBMLE not only outperforms existing methods but also adapts well to datasets of varying scales and complexities, making it a robust and generalizable classifier.

### 4.5. TAN-FGBMLE for Graph Structure Learning on Air Quality Data

After validating TAN-FGBMLE on benchmark classification datasets, we further explore its applicability in domain-specific real-world data by a graph structure learning experiment on the open-source air quality dataset. This dataset contains daily records of multiple continuous environmental variables such as temperature, humidity, and pollutant concentrations including NO_2_, SO_2_, CO, PM2.5, and PM10, providing a representative test case for evaluating TAN structure learning.

**Figure 7:**
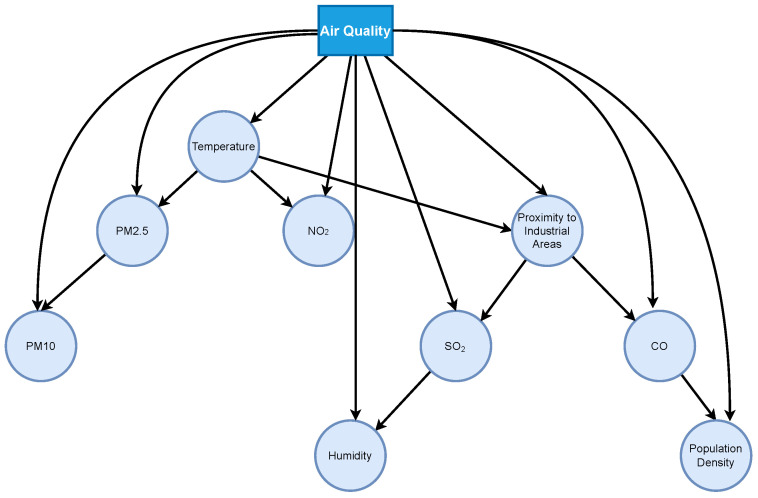
Dependency structure diagram of the AirQuality dataset based on Prim’s algorithm.

The dependency tree is shown in Figure 7. The figure shows a direct connection between PM2.5 and PM10, indicating a high correlation between the two. The edge relationship between temperature and NO_2_ reveals the influence of meteorological conditions on nitrogen oxide concentrations. The connection between industrial proximity and SO_2_ and CO demonstrates the dominant role of emission sources. The relationship between CO and population density reflects the contribution of human activities to air quality. These findings are consistent with the generation mechanism of atmospheric pollutants and demonstrate that the Prim algorithm can extract a reasonable and interpretable dependency structure.

After constructing the TAN structure, we further compared the edge stability performance under different density estimation methods. We used bootstrap sampling to repeatedly estimate the mutual information and counted the frequency of occurrence of key dependency edges across repeated experiments, denoting this as the Bootstrap Edge Consistency (BEC) metric [50] . A higher BEC value indicates that the edge can be stably identified under different sampling conditions, demonstrating the robustness of the structure learning. Its mathematical definition is $BEC\left(e_{i,j}\right)=\frac{1}{B}\sum_{b=1}^{B}\delta_{i,j}^{\left(b\right)}$, where $\delta_{ij}^{\left(b\right)}=1$ if edge $e_{i,j}$ appears in the *b*th bootstrap result, and 0 otherwise. A higher BEC value indicates that the edge is more stable under different sampling conditions. The dependency structure is more explanatory.

Table 8 shows the BEC comparison results for some key edges. It can be seen that the traditional KED is unstable in estimating mutual information in multimodal and heteroskedastic data, resulting in low edge frequency. Our method TAN-FGBMLE exhibits higher BEC values for all key dependency edges. For example, the PM2.5–PM10 relationship improves from 0.71 to 0.92, and the temperature–NO_2_ relationship improves from 0.64 to 0.88. These results demonstrate that TAN-FGBMLE not only improves stability in structure learning on real-world air quality data but also provides interpretable insights into environmental and pollution-related dependencies, highlighting its potential for practical applications beyond benchmark datasets.

### 4.6. Summary of Strengths and Limitations

The experimental analyses above show that the proposed FGBMLE framework performs particularly well in scenarios involving complex, multimodal, or high-dimensional data distributions. By decoupling the candidate generation and mixture reweighting processes, FGBMLE effectively captures intricate latent structures while maintaining stability under limited-sample conditions. Compared with classical EM-based or kernel density methods, it consistently achieves smoother likelihood surfaces, lower estimation bias, and improved robustness in conditional mutual information computation and TAN structure learning.

Nevertheless, the method may underperform when the underlying data distribution is simple and unimodal—such as Gaussian-like datasets—where the flexibility of the generator offers limited additional benefit while increasing computational cost. Furthermore, in extremely high-dimensional cases with very small sample sizes, the Stage-II reweighting process may exhibit instability due to insufficient density support. In such cases, conventional parametric models or regularized variants of FGBMLE may yield more stable performance. Future work will explore adaptive regularization and model selection strategies to mitigate these limitations and extend the framework’s scalability to ultra-high-dimensional domains.

## 5. Conclusions

This paper addresses the difficulty of accurately estimating the class-conditional mutual information in the TAN model under continuous attribute conditions. We propose an improved method, TAN-FGBMLE, based on the FGBMLE framework. This method efficiently models complex mixture densities by introducing a generative network and a two-stage optimization framework, significantly improving computational efficiency while maintaining estimation accuracy. Experimental results show that TAN-FGBMLE outperforms traditional nonparametric methods in classification with multiple public datasets and real-world air quality data. The learned graph structure reflects the dependencies between continuous attributes, demonstrating strong interpretability. Current applications focus primarily on classification tasks, and the method’s applicability to other fields requires further verification. Future research could focus on optimizing the network structure, enhancing its ability to characterize complex distributions, and expanding its application to scenarios such as time series analysis and anomaly detection.

## Figures and Tables

**Table 1:** Simulation scenarios of the FGBMLE method.

Distribution	Gaussian Mixture Model (GMM)	Gamma Mixture Model (GaMM)
*d-dimension*	$\pi \left(\theta \right)=0.5\;N\left(\mu_{1},\Sigma_{1}\right)+0.5\;N\left(\mu_{2},\Sigma_{2}\right)$	$\pi \left(\theta \right)=\prod_{j=1}^{d}\mathrm{Beta}\left(10,5\right)$
	$x|\theta ∼N(\theta ,I_{d})$	$x|\theta ∼\prod_{j=1}^{d}\mathrm{Gamma}\left(10,\theta_{j}\right)$
	$\mu_{1}={\left(-3,-3,\ldots ,-3\right)}^{⊤}\in R^{d}$	
	$\mu_{2}={\left(3,3,\ldots ,3\right)}^{⊤}\in R^{d}$	
	$\Sigma_{1}=2I_{d},\;\Sigma_{2}=I_{d}$	

**Table 2:** Performance comparison of different methods under two-dimensional simulation scenarios.

Model	Method	W1	ISE	MSE	KL
GMM	FGBMLE	0.335	0.009	0.006	0.045
	Bootstrap	0.310	0.010	0.008	0.067
	KDE	0.482	0.026	0.014	0.112
GaMM	FGBMLE	0.035	0.270	0.011	0.083
	Bootstrap	0.038	0.510	0.014	0.094
	KDE	0.072	0.693	0.023	0.141

**Table 3:** Density estimation results of different methods under a trimodal distribution.

Method	W1	ISE	MSE	KL
KDE	0.154	0.0314	0.0125	0.0847
GMM (K=2)	0.102	0.0246	0.0097	0.0632
GMM (K=3)	0.068	0.0179	0.0064	0.0415
TAN-FGBMLE	0.041	0.0103	0.0048	0.0286

**Table 4:** Average SHD values of different methods under two sample sizes.

Method	SHD (*n* = 1000)	SHD (*n* = 2000)
TAN-KDE	0.4 ± 0.5	0.2 ± 0.4
TAN-GMM	0.5 ± 0.4	0.3 ± 0.6
**TAN-FGBMLE**	**0.3 ± 0.4**	**0.2 ± 0.2**

**Table 5:** Performance comparison of TAN-FGBMLE and competing models on the simulated dataset (n=2000). Statistical significance is based on the Wilcoxon signed-rank test at α=0.05.

Model	Accuracy (%)	Log-Likelihood
NB	83.5 ± 1.2	−2.37 ± 0.08
TAN	86.8 ± 0.9	−2.13 ± 0.07
AODE	87.4 ± 0.8	−2.09 ± 0.05
WAODE	87.9 ± 0.8	−2.07 ± 0.05
HNB	88.1 ± 0.7	−2.02 ± 0.05
CFWNB	88.5 ± 0.6	−1.98 ± 0.04
KDB-2	88.6 ± 0.6	−1.97 ± 0.04
Logistic Regression	88.9 ± 0.6	−1.95 ± 0.04
Random Forest	90.2 ± 0.5	−1.89 ± 0.03
RBF-SVM	89.8 ± 0.5	−1.90 ± 0.04
**TAN-FGBMLE**	**91.1 ± 0.4**	**−1.82 ± 0.03**

**Table 6:** Description of the datasets used in experiments.

No.	Dataset	Instances	Attributes	Classes
1	Abalone	4177	8	3
2	Breast Cancer	569	30	2
3	Car Evaluation	1728	6	4
4	Credit Approval	690	15	2
5	Dermatology	366	34	6
6	*E. coli*	336	7	8
7	Glass	214	9	6
8	Haberman	306	3	2
9	Heart Disease	303	13	2
10	ILPD	583	9	2
11	Ionosphere	351	34	2
12	Iris	150	4	3
13	Landsat Satellite	2000	36	6
14	Parkinsons	195	22	2
15	Pima Indians Diabetes	768	8	2
16	Student Performance	649	33	2
17	Vehicle	846	18	4
18	Wine	178	13	3
19	Wine Quality	1599	11	10
20	Yeast	1484	8	10

**Table 7:** Classification accuracy comparison of the proposed method against existing classifiers.

Dataset Name	TAN-KDE	NBC	FBC	KNN	C4.5	NN	SVM	TAN-FGBMLE
Abalone	0.512 ± 0.067	0.498 ± 0.058	0.505 ± 0.061	0.528 ± 0.064	0.490 ± 0.055	0.545 ± 0.060	0.551 ± 0.066	**0.612** ± 0.059
Breast Cancer	0.861 ± 0.042	0.845 ± 0.046	0.850 ± 0.039	0.872 ± 0.040	0.858 ± 0.044	0.875 ± 0.036	0.868 ± 0.042	**0.889** ± 0.038
Car Evaluation	0.902 ± 0.035	0.890 ± 0.033	0.896 ± 0.034	0.918 ± 0.037	0.910 ± 0.038	0.922 ± 0.032	0.916 ± 0.036	**0.934** ± 0.031
Credit Approval	0.823 ± 0.047	0.812 ± 0.042	0.818 ± 0.043	0.834 ± 0.041	0.826 ± 0.048	0.842 ± 0.039	0.838 ± 0.044	**0.856** ± 0.037
Dermatology	0.931 ± 0.028	0.920 ± 0.030	0.925 ± 0.027	0.936 ± 0.031	0.929 ± 0.029	0.942 ± 0.025	0.938 ± 0.028	**0.951** ± 0.023
*E. coli*	0.825 ± 0.055	0.810 ± 0.052	0.814 ± 0.050	0.838 ± 0.048	0.822 ± 0.053	0.846 ± 0.047	0.840 ± 0.051	**0.862** ± 0.046
Glass	0.673 ± 0.071	0.652 ± 0.074	0.660 ± 0.070	0.684 ± 0.072	0.676 ± 0.068	0.691 ± 0.066	0.687 ± 0.071	**0.712** ± 0.065
Haberman	0.738 ± 0.058	0.720 ± 0.061	0.728 ± 0.057	0.741 ± 0.060	0.732 ± 0.062	0.752 ± 0.054	0.746 ± 0.059	**0.764** ± 0.053
Heart Disease	0.836 ± 0.046	0.820 ± 0.048	0.828 ± 0.044	0.844 ± 0.047	0.832 ± 0.045	0.850 ± 0.041	0.846 ± 0.046	**0.868** ± 0.040
ILPD	0.752 ± 0.062	0.740 ± 0.059	0.746 ± 0.061	0.758 ± 0.057	0.749 ± 0.060	0.763 ± 0.055	0.760 ± 0.058	**0.778** ± 0.053
Ionosphere	0.887 ± 0.036	0.872 ± 0.039	0.880 ± 0.035	0.894 ± 0.038	0.885 ± 0.037	0.901 ± 0.033	0.896 ± 0.036	**0.912** ± 0.032
Iris	0.955 ± 0.028	0.940 ± 0.032	0.948 ± 0.029	0.958 ± 0.027	0.950 ± 0.030	0.962 ± 0.026	0.959 ± 0.028	**0.970** ± 0.025
Landsat Satellite	0.704 ± 0.065	0.688 ± 0.068	0.694 ± 0.064	0.716 ± 0.066	0.707 ± 0.067	0.724 ± 0.061	0.719 ± 0.065	**0.738** ± 0.060
Parkinsons	0.823 ± 0.051	0.810 ± 0.054	0.816 ± 0.050	0.829 ± 0.052	0.820 ± 0.053	0.834 ± 0.049	0.828 ± 0.052	**0.846** ± 0.047
Pima Indians Diabetes	0.775 ± 0.059	0.762 ± 0.061	0.770 ± 0.058	0.782 ± 0.056	0.774 ± 0.060	0.788 ± 0.055	0.784 ± 0.058	**0.802** ± 0.054
Student Performance	0.741 ± 0.064	0.728 ± 0.067	0.734 ± 0.062	0.748 ± 0.066	0.739 ± 0.065	0.755 ± 0.060	0.750 ± 0.063	**0.768** ± 0.058
Vehicle	0.746 ± 0.057	0.730 ± 0.060	0.738 ± 0.056	0.751 ± 0.059	0.742 ± 0.058	0.756 ± 0.054	0.752 ± 0.057	**0.770** ± 0.052
Wine	0.944 ± 0.030	0.930 ± 0.033	0.938 ± 0.029	0.948 ± 0.031	0.940 ± 0.032	0.952 ± 0.027	0.949 ± 0.030	**0.960** ± 0.026
Wine Quality	0.706 ± 0.062	0.691 ± 0.065	0.698 ± 0.061	0.711 ± 0.064	0.703 ± 0.063	0.718 ± 0.059	0.714 ± 0.062	**0.732** ± 0.058
Yeast	0.602 ± 0.071	0.586 ± 0.074	0.592 ± 0.069	0.608 ± 0.072	0.598 ± 0.070	0.614 ± 0.066	0.610 ± 0.071	**0.628** ± 0.065
Average	0.782 ± 0.054	0.768 ± 0.057	0.774 ± 0.053	0.789 ± 0.056	0.780 ± 0.055	0.795 ± 0.051	0.791 ± 0.054	**0.812** ± 0.049

**Table 8:** Comparison of BEC of critical edges.

Edge (Undirected)	TAN-KDE	TAN-FGBMLE
PM2.5–PM10	0.71	0.92
temperature–NO_2_	0.64	0.88
Proximity to industrial areas–SO_2_	0.62	0.85
Proximity to industrial areas–CO	0.58	0.81
humidity–PM2.5	0.49	0.74
CO–population density	0.56	0.79

## Data Availability

The data that support the findings of this study are available in the UCI dataset https://archive.ics.uci.edu/datasets (accessed on 24 October 2025). The code of the paper is in the following link: https://github.com/ZTY0516/TAN-FGBMLE (accessed on 24 October 2025).
